# Evaluating the oxysterol combination of 22(S)-hydroxycholesterol and 20(S)-hydroxycholesterol in periodontal regeneration using periodontal ligament stem cells and alveolar bone healing models

**DOI:** 10.1186/s13287-017-0725-9

**Published:** 2017-12-06

**Authors:** Jin-Sun Lee, EunJi Kim, Seonggu Han, Kyung Lhi Kang, Jung Sun Heo

**Affiliations:** 1 Department of Maxillofacial Biomedical Engineering and Institute of Oral Biology, School of Dentistry, Kyung Hee University, 26 Kyunghee-daero, Dongdaemun-gu, Seoul 02447 South Korea; 2 Department of Periodontology, School of Dentistry, Kyung Hee University, 26 Kyunghee-daero, Dongdaemun-gu, Seoul 02447 South Korea

**Keywords:** Oxysterol, Periodontal ligament stem cells, Osteogenic differentiation, Alveolar bone defect, Bone regeneration

## Abstract

**Background:**

Oxysterols, oxygenated by-products of cholesterol biosynthesis, play roles in various physiological and pathological systems. However, the effects of oxysterols on periodontal regeneration are unknown. This study investigated the effects of the specific oxysterol combination of 22(S)-hydroxycholesterol and 20(S)-hydroxycholesterol (SS) on the regeneration of periodontal tissues using in-vitro periodontal ligament stem cells (PDLSCs) and in-vivo models of alveolar bone defect.

**Methods:**

To evaluate the effects of the combined oxysterols on PDLSC biology, we studied the SS-induced osteogenic differentiation of PDLSCs by assessing alkaline phosphatase activity, intracellular calcium levels [Ca^2+^]_i_, matrix mineralization, and osteogenic marker mRNA expression and protein levels. To verify the effect of oxysterols on alveolar bone regeneration, we employed tooth extraction bone defect models.

**Results:**

Oxysterols increased the osteogenic activity of PDLSCs compared with the control group. The expression of liver X receptor (LXR) α and β, the nuclear receptors for oxysterols, and their target gene, ATP-binding cassette transporter A1 (*ABCA1*), increased significantly during osteogenesis. Oxysterols also increased protein levels of the hedgehog (Hh) receptor Smo and the transcription factor Gli1. We further confirmed the reciprocal reaction between the LXRs and Hh signaling. Transfection of both LXRα and LXRβ siRNAs decreased Smo and Gli1 protein levels. In contrast, the inhibition of Hh signaling attenuated the LXRα and LXRβ protein levels. Subsequently, SS-induced osteogenic activity of PDLSCs was suppressed by the inhibition of LXRs or Hh signaling. The application of SS also enhanced bone formation in the defect sites of in-vivo models, showing equivalent efficacy to recombinant human bone morphogenetic protein-2.

**Conclusions:**

These findings suggest that a specific combination of oxysterols promoted periodontal regeneration by regulating PDLSC activity and alveolar bone regeneration.

**Electronic supplementary material:**

The online version of this article (doi:10.1186/s13287-017-0725-9) contains supplementary material, which is available to authorized users.

## Background

Periodontal regeneration reconstructs the tooth’s supporting periodontal tissues, such as the periodontal ligament, cementum, and alveolar bone, following damage due to periodontal disease or dental defects [1]. Although nonsurgical treatment and periodontal surgery usually yield successful clinical outcomes, healing after these therapeutic approaches is characterized by the formation of a junctional epithelium along the root surfaces, without periodontal tissue renewal [2]. The biological rationale for periodontal regeneration is based on one of the following mechanisms: activation of bone-forming cells (i.e., stem cells, periodontal ligament cells, and osteoblasts); materials for periodontal tissue formation (i.e., bone graft substitutes); or osteoinductive factors [3]. Given the complex composition of the periodontal tissue, regeneration analysis must be confirmed using validated models. Thus, the present study employed in-vitro and in-vivo compartmentalized systems: periodontal ligament stem cell (PDLSC) activity screening; and experimentally created alveolar bone defect models to verify the periodontium regeneration using various phases.

PDLSCs are important for the homeostasis of the periodontium. These stem cells are regarded not only as putative candidates for dental tissue engineering [4, 5], but also as a potential resource to differentiate into bone-forming osteoblasts [6], which could be useful for the reconstruction of damaged periodontium and bone tissues. Furthermore, PDLSCs can be easily collected from baby teeth and wisdom teeth, unlike harvesting bone marrow-derived stem cells by invasive surgery or cord blood stem cells at birth. Thus, PDLSCs are promising models to study the healing of the orofacial region, which comprises both soft and hard tissues.

Bone tissue regeneration is a complex healing event involving many biological modulators, such as growth factors, extracellular matrix proteins, and attachment factors [7]. Bone regeneration-related growth factors are biologically active peptides or proteins that influence the proliferation and differentiation of bone-forming tissue cells [8]. Among these factors, bone morphogenetic protein‐2 (BMP‐2) is frequently used to encourage bone formation. Although BMP‐2 exerts the most potent osteoinductive effects, safety concerns and associated complications including inflammation, ectopic bone formation, bone resorption, and carcinogenesis have been reported [9, 10]. To date, functional alternatives that lack side effects have not been described in detail.

Previously, we reported that a combination of specific oxysterols promoted the osteogenic differentiation of embryonic stem cells (ESCs) and showed a similar efficacy to BMP-2 [11]. Oxysterols are oxygenated by-products of cholesterol biosynthesis that are present naturally in healthy human or animal tissues. Currently, these molecules are believed to act as endogenous modulators in lipid metabolism and other biological activities [12]. Previous studies demonstrated the potential osteogenic role of oxysterols in the osteogenic differentiation of stem cells and in-vivo bone formation [13–15]. Treatment with a single oxysterol, such as 20(S)‐hydroxycholesterol (20S), adequately induced osteogenic and anti‐adipogenic effects in multipotent mesenchymal stem cells (MSCs) [16]. In addition, particular combinations of the 22(R)-hydroxycholesterol or 22(S)-hydroxycholesterol and 20(S)-hydroxycholesterol oxysterols enhanced the osteogenesis of MSCs, in which critical signaling pathways were involved [17–19]. Thus, oxysterols are naturally occurring and cost-effective osteogenic factors that could act as substitutes for BMP-2 in the dental and orthopedic fields.

The present study assessed the potency of oxysterols in the regeneration of periodontal tissues using well-designed approaches comprising in-vitro PDLSC activity assays and in-vivo alveolar bone defect models.

## Methods

### Materials

Fetal bovine serum (FBS) was purchased from Gibco-BRL (Gaithersburg, MD, USA). Anti-COLIA, OPN, OCN, OSX, RUNX2, LXRα, LXRβ, Smo, Gli1, β-actin, goat anti-mouse, and goat anti-rabbit antibodies were supplied by Santa Cruz Biotechnology (Santa Cruz, CA, USA). Unless otherwise specified, chemicals and laboratory wares were from the Sigma Chemical Company (St Louis, MO, USA) and Falcon Labware (Becton-Dickinson, Franklin Lakes, NJ, USA), respectively.

### Periodontal ligament stem cell culture

Periodontal ligaments were obtained from extracted human molars donated by the Kyung Hee University Department of Oral and Maxillofacial Surgery. All subjects involved in this study were informed about its purpose and procedures, and the study was approved by the Kyung Hee University Review Board. Written informed consent was obtained from all donors or their guardians on behalf of minor participants.

Periodontal ligaments were collected from the middle thirds of roots and cultured in α-minimal essential medium (α-MEM; Gibco-BRL) containing 10% FBS, penicillin (100 U/ml), and streptomycin (100 μg/ml; Gibco-BRL), according to a method described previously [20, 21]. After two passages, the cells were subjected to magnetic isolation using magnetic beads (Miltenyi Biotec, Germany) and antibodies to detect the STRO-1 antigen (mesenchymal stem cell marker; Millipore, Billerica, MA, USA). The resulting STRO-1(+) cell population was cultured in α-MEM plus 10% FBS at 37 °C with a humidified gas mixture of 5% CO_2_/95% air. All experiments were carried out with passage 4–7 cells. To induce osteogenic differentiation, cells were maintained in an osteogenic medium comprised of α-MEM containing 5% FBS, 50 μg/ml ascorbic acid, 1 μM dexamethasone, and 3 mM β-glycerophosphate for 1 day before the application of oxysterols (22(S)-hydroxycholesterol and 20(S)-hydroxycholesterol (SS)). Different concentrations of oxysterols (0.5–5 μM) were supplemented into the osteogenic medium, which was changed every other day. Oxysterols were dissolved in dimethyl sulfoxide (DMSO) immediately before use, and the final concentration of DMSO did not exceed 0.1% (v/v) in any of the experiments. DMSO at 0.1% was used as a control.

### Alkaline phosphatase activity

Alkaline phosphatase (ALP) activity was assayed as described previously [21]. Briefly, the cells were washed twice with phosphate-buffered saline (PBS) and lysed in 50 mM Tris–HCl buffer (pH 7.0) containing 1% (v/v) Triton™ X-100 and 1 mM phenylmethylsulfonyl fluoride (PMSF). The total protein concentration was then quantified according to the Bradford method [22]. The entire cell lysate was assayed by adding 200 μl *p*-nitrophenylphosphate (Sigma Chemical Company) as a substrate for 30 min at 37 °C. The reaction was stopped by the addition of 3 M NaOH and the absorbance was read on a spectrophotometer at 405 nm. The enzyme activity was expressed as millimoles per 100 μg of protein.

### Intracellular calcium assay

The cells were washed three times with PBS and lysed in 50 mM Tris–HCl buffer (pH 7.0) containing 1% (v/v) Triton™ X-100 and 1 mM PMSF without EDTA. The protein content was then quantified according to the Bradford method [22]. The intracellular calcium content was measured using a calcium assay kit according to the manufacturer’s instructions (BioAssay Systems, Hayward, CA, USA.), and the absorbance was read on a spectrophotometer at 602 nm. The calcium content was expressed as milligrams per 100 mg of protein.

### Alizarin Red staining

Alizarin Red staining was performed as described in our previous publication [23]. Briefly, the culture media were discarded and cells were fixed for 20 min in 4% paraformaldehyde, washed three times with ice-cold PBS, and stained for 20 min with Alizarin Red (pH 4.2; Sigma). Finally, the solution was aspirated, and the cells were washed with deionized water and then observed under a light microscope. To quantify mineralization, bound dye was extracted in 10 mM sodium phosphate containing 10% cetylpyridinium chloride and quantified spectrophotometrically at 562 nm.

### RNA isolation and real-time reverse-transcriptase polymerase chain reaction

Real-time reverse-transcriptase polymerase chain reaction (RT-qPCR) was performed as described in our previous study [21]. Total RNA was extracted from the cells using the TRIzol™ reagent (Invitrogen, USA) following the manufacturer’s protocol. Real-time quantification of RNA targets was then performed using a Rotor-Gene 2000 real-time thermal cycling system (Corbett Research, Australia) with a QuantiTect SYBR® Green reverse-transcriptase polymerase chain reaction (RT-PCR) kit (Qiagen, CA, USA). The reaction mix (20 μl) contained 200 ng of total RNA, 0.5 μM of each primer, and appropriate amounts of enzymes and fluorescent dyes, as recommended by the supplier. The Rotor-Gene 2000 cycler was programmed as follows: 30 min at 50 °C for reverse transcription, 15 min at 95 °C for DNA polymerase activation, 15 s at 95 °C for denaturing, followed by 45 cycles of 15 s at 94 °C, 30 s at 55 °C, and 30 s at 72 °C. Data were collected during the extension step (30 s at 72 °C). The PCR reaction was followed by melting curve analysis to verify the specificity and identity of the RT-qPCR products, which distinguished specific PCR products from nonspecific PCR products resulting from primer dimer formation. The temperature of the PCR products was increased from 65 °C to 99 °C at a rate of 1 °C/5 s, and the resulting data were analyzed using the manufacturer’s software. The primers used were 5′-TGA AAC GAG TCA GCT CTG GAT G-3′ (forward) and 5′-TGA AAT TCA TGG CTG TGG AA-3′ (reverse) for *OPN*; 5′-TGA GGA GGA AGT TCA CTA TGG-3′ (forward) and 5′-TTC TTT GTG CCT GCT TTG C-3′ (reverse) for *OSX*; 5′-ATG AGA GCC CTC ACA CTC CTC-3′ (forward) and 5′-GCC GTA GAA GCG CCG ATA GGC-3′ (reverse) for *OCN*; 5′-AAG CCC ATG CCT ACG T-3′ (forward) and 5′-TGC AGA CGC AGT GCA AAC A-3′ (reverse) for *LXRα*; 5′-TCG TGG ACT TCG CTA AGC AA-3′ (forward) and 5′-GCA GCA TGA TCT CGA TAG TGG A-3′ (reverse) for *LXRβ*; 5′-CCC TGT GGA ATG TAC CTA TGT G-3′ (forward) and 5′-GAG GTG TCC CAA AGA TGC AA-3′ (reverse) for *ABCA1*; and 5′-GCT CTC CAG AAC ATC ATC C-3′ (forward) and 5′-TGC TTC ACC ACC TTC TTG-3′ (reverse) for *GAPDH*.

### Western blotting analysis

Western blotting analysis was conducted as reported previously [21]. Protein extract samples (20 μg) were separated by 8–10% sodium dodecyl sulfate polyacrylamide gel electrophoresis and blotted onto polyvinylidene difluoride membranes. The blots were washed with TBST (10 mM Tris–HCl (pH 7.6), 150 mM NaCl, 0.05% Tween-20), blocked with 5% skim milk for 1 h, and incubated with the appropriate primary antibodies (anti-OCN, anti-OSX, anti-Runx2, anti-COLA1, anti-ALP, anti-LXR α, anti-LXR β, anti-SMO, anti-Gli1, or anti-β-actin; Santa Cruz Biotechnology) at the dilutions recommended by the supplier. The membranes were then washed and the primary antibodies were detected with goat anti-rabbit immunoglobulin G (IgG) or goat anti-mouse IgG conjugated to horseradish peroxidase. The blots were developed with enhanced chemiluminescence (Bio-Rad Laboratories, Hercules, CA, USA) and exposed to X-ray film (Eastman-Kodak, Rochester, NY, USA).

In the experiments involving animal tissues, the specimens containing sockets were acquired from the maxilla using a trephine bur (inner diameter 3.3 mm, outer diameter 4.0 mm) (XTP3404; Dentium, Seoul, Korea) after the rats were euthanized. The specimens were immediately stored at −80 °C and ground with a mortar and pestle. The ground specimens were lysed in ice-cold radioimmunoprecipitation assay buffer (RIPA buffer; Cell Signaling, Danvers, MA, USA) containing 1 mM PMSF and clarified by centrifugation at 12,000 rpm for 20 min. Protein concentrations were determined using a protein assay kit (Bio-Rad Laboratories). Western blotting analysis was then performed as already described.

### Immunofluorescence staining

The cells were washed with PBS, fixed for 5 min using 4% paraformaldehyde, and permeated for 20 min using 0.1% Triton™ X-100 at room temperature. The cells were then washed three times and blocked for 1 h using 4% BSA in PBS at room temperature. The cells were treated with primary antibodies (1:100; rabbit anti-OCN, rabbit anti-OSX, rabbit anti-Runx2) and incubated overnight at 4 °C. Subsequently, the cells were treated with Alexa Fluor® 488 or 594 goat anti-rabbit IgG (1:500; Invitrogen Life Technologies, USA) for 2 h at room temperature. Fluorescence images were obtained under a fluorescence microscope (Fluoview 300; Olympus, Japan).

### siRNA transfection

Cells were transfected for 24 h with a Stealth small interfering RNA (siRNA) specific to *LXRα*, *LXRβ* (100 nM; Bioneer, Korea), or an unrelated control siRNA targeting the green fluorescent protein. The sequences of the siRNAs were as follows: 5′-GAG ACA UCU CGG AGG UAC A-3′ (forward) and, 5′-UGU ACC UCC GAG AUG UCU C-3′ (reverse) for siRNA-LXRα; and 5′-CGA GCU UUG CCG UGU CUG U-3′ (forward) and 5′-ACA GAC ACG GCA AAG CUC G-3′ (reverse) for siRNA-LXRβ. Briefly, the cells were seeded and grown in 60-mm culture dishes until they reached 70% confluence. The serum was then exchanged with antibiotic-free media and incubation continued for 24 h. The cells were transfected for 24 h with either an siRNA specific to LXR or a negative control siRNA (scrambled) targeting a site that is absent from the human, mouse, and rat genomes using the Lipofectamine® RNAiMAX transfection reagent (Invitrogen, USA) according to the manufacturer’s instructions, before being subjected to chemical treatments.

### Alveolar bone defect model and study design

**Fig. 1:**
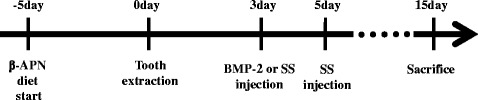
Critical steps in the animal experiment. Timeline for establishment of the alveolar bone defect model and the injection of each agent. β-APN β-aminopropionitrile, BMP‐2 bone morphogenetic protein‐2, SS 22(S)-hydroxycholesterol and 20(S)-hydroxycholesterol

Eighteen 8-week-old male Sprague–Dawley rats weighing 250–300 g were used for this study. The rats were divided into three groups: the control, BMP-2, and SS groups. Each group contained six rats, three for western blotting analysis and the other three for micro-computed tomography (μCT) analysis. After extraction of the left and right maxillary first molars from each rat, the extraction sockets were left untreated for natural healing in the control group. Recombinant human bone morphogenetic protein-2 (rhBMP-2; Cowellmedi Co., Ltd, Seoul, Korea) was injected into both extraction sockets on the third day after the extraction in the BMP-2 group. Also, SS was injected twice, on the third and fifth days after the extraction, in the SS group (Fig. 1). All rats were provided food and water ad libitum, and were housed under standardized environmental conditions. The rats were euthanized 15 days after extraction via asphyxiation in a CO_2_ chamber. All experimental procedures were approved by the Institutional Animal Care and Use Committee of Kyung Hee University Hospital at Gangdong (KHNMC AP 2016-002).

### Tooth extraction

For 5 days before the extraction procedure, the rats were fed a diet containing β-aminopropionitrile (β-APN; Sigma-Aldrich, St Louis, MO, USA) at a rate of 0.4% β-APN per gram of chow to facilitate subsequent extraction of the maxillary first molars. Under general anesthetic via an intramuscular injection of alfaxalone (Alfaxan®, 0.1 ml/100 g), the left and right maxillary first molars of all rats (*n* = 48) were extracted, and bleeding was controlled. An intramuscular injection of gentamicin (3 mg/kg; DaeSung Microbiological Labs, Uiwang, Korea) and a subcutaneous injection of 1% ketoprofen (0.3 ml/kg; Uni Biotech, Chungnam, Korea) were administered to prevent infection and to relieve pain, respectively, for 3 days after the extraction.

### Injection of oxysterols and rhBMP-2

The oxysterol combination (SS) was prepared by mixing equal amounts of 100 μM 20S-hydroxycholesterol (Sigma-Aldrich) with 100 μM 22S-hydroxycholesterol (Sigma-Aldrich) dissolved in 1% DMSO in PBS. The other test agent, rhBMP-2, was diluted to a saturated concentration of 1.5 mg/ml with injectable saline. A 50-μl Hamilton syringe (no. 705; Hamilton Company, Reno, NV, USA) with a 32-gauge needle was used to administer an injection into the extraction sockets. Under general anesthesia, 10 μl of SS and 10 μl of rhBMP-2 were injected slowly into the extraction sockets of the rats in each group.

### Micro-computed tomography

All specimens were fixed in 10% formalin for 7 days and then imaged using a high-resolution μCT system (Skyscan 1173; Skyscan, Kontich, Belgium) at 90 kV and 88 μA, with an image pixel size of 9.94 μm. The regions of interest (ROI) were defined as five-root sockets of each tooth from the alveolar crest to the apex. The scanned data for the ROI were reconstructed and analyzed using three-dimensional analysis software (NRecon software; Skyscan). Newly formed bone volume (%) was calculated by multiplying the quotient of the bone volume divided by the total volume of the ROI by 100.

### Statistical analysis

All data are expressed as the mean ± standard deviation (SD). One-way ANOVA was used for multiple comparisons (Duncan’s multiple range test), using SPSS software version 10.0. *P* < 0.05 was considered statistically significant. The animal study data were analyzed using the nonparametric Kruskal–Wallis test, and by the Tukey HSD test for multiple comparisons (SAS for Windows version 9.2). *P* < 0.05 was considered statistically significant.

## Results

### Effect of oxysterols on PDLSC osteogenic differentiation

**Fig. 2:**
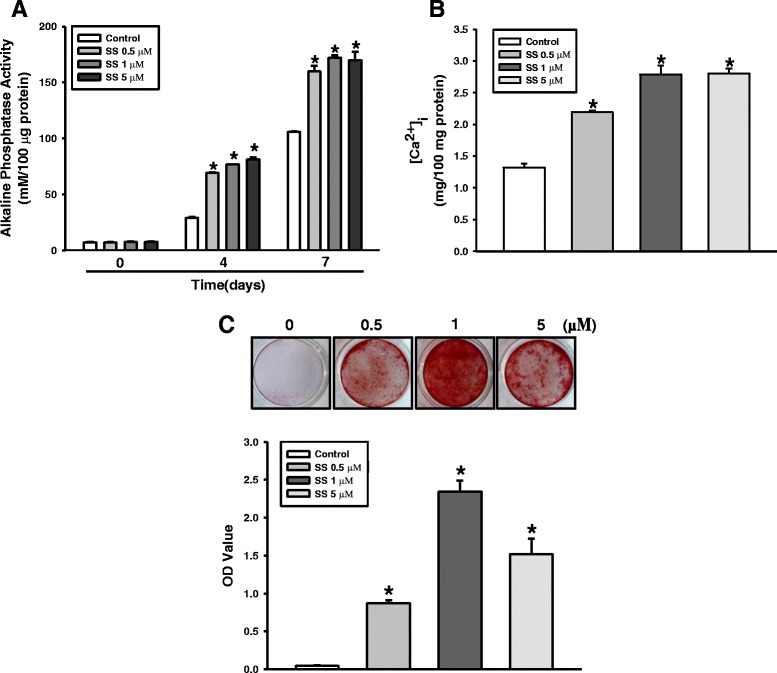
Effect of oxysterols on PDLSC osteogenesis. Cells treated with different oxysterol concentrations (SS; 0.5, 1, and 5 μM) for 0, 4, 7, or 14 days. **a** ALP activity, **b** [Ca^2+^]_i_, and **c** Alizarin red staining. Lower panels (bars) denote Alizarin Red quantification. Values reported as means ± SD of five independent experiments. *P < 0.05 vs control value at each time point. [Ca^2+^]_i_ intracellular calcium level, SS 22(S)-hydroxycholesterol and 20(S)-hydroxycholesterol

**Fig. 3:**
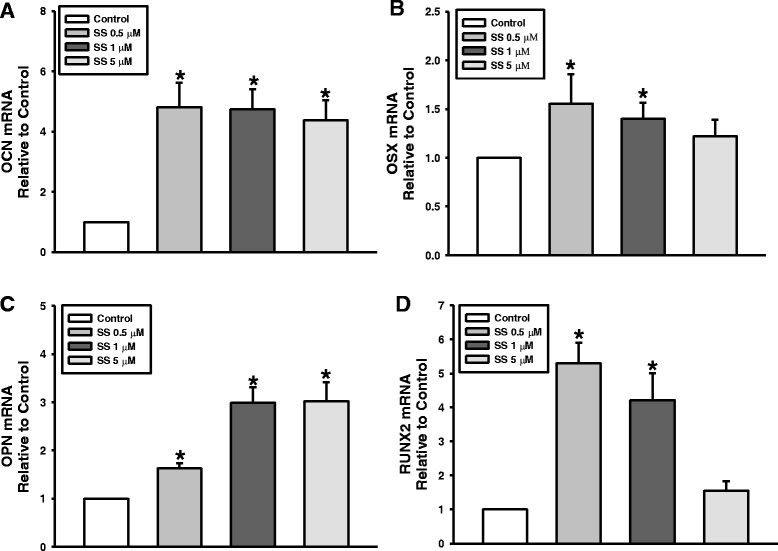
Effect of oxysterols on osteogenic gene expression. mRNA levels of **a** *OCN*, **b** *OSX*, **c** *OPN*, and **d** *RUNX2* analyzed after 7 days of osteogenic induction. Values reported as means ± SD of five independent experiments. *P < 0.05 vs control value. OCN osteocalcin, OPN osteopontin, OSX osterix, RUNX2 Runt-related transcription factor 2

**Fig. 4:**
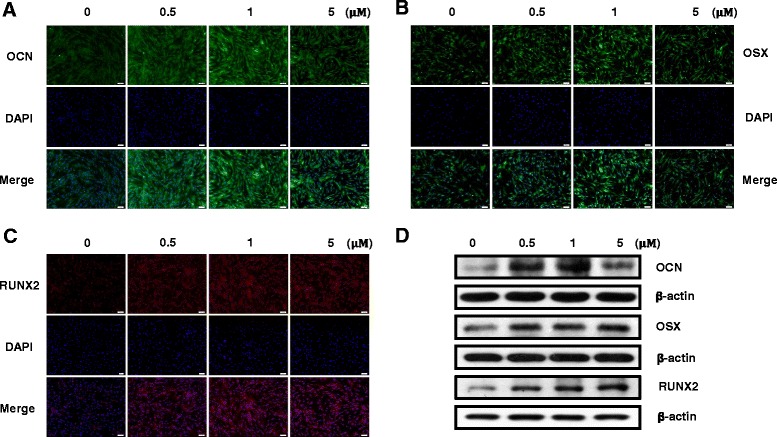
Effect of oxysterols on osteogenic-related protein levels. Protein levels of OCN, OSX, and RUNX2 determined by (**a–c**) immunofluorescence staining and **(d)** western blotting (OCN 5.5 kDa, OSX 45 kDa, RUNX2 55 kDa). Nuclei stained with DAPI (blue). Representative result from three independent experiments (scale bar, 100 μm). DAPI 4′,6-diamidino-2-phenylindole, OCN osteocalcin, OSX osterix, RUNX2 Runt-related transcription factor 2

To verify the osteogenic effect of oxysterols on PDLSCs, the cells were exposed to 0.5, 1, and 5 μM of the SS oxysterol cocktail for 0, 4, 7, and 14 days. ALP activity, intracellular calcium levels [Ca^2+^]_i_, and mineralization were then determined. Figure 2a–c shows that ALP activity, [Ca^2+^]_i_, and mineralization increased significantly in PDLSCs exposed to 0.5, 1, and 5 μM of SS compared with the control groups. To characterize the osteogenic differentiation further, we assessed the mRNA expression of the osteogenic target genes *OCN*, *OSX*, *OPN*, and *RUNX2* using RT-qPCR. The expression did not increase dose dependently; however, cells treated with SS demonstrated higher gene expression compared with the control groups (Fig. 3a–d). We also determined the osteogenic effect of oxysterols on PDLSCs by assessing the protein levels of the osteogenic markers OCN, OSX, and RUNX2 at day 7 of osteogenic induction. Immunofluorescence staining for the osteogenic markers confirmed that SS treatment promoted PDLSC osteogenesis (Fig. 4a–c). Western blotting analysis also showed that the OCN, OSX, and RUNX2 levels increased in the cultures treated with SS compared with the control groups (Fig. 4d).

### Involvement of LXRs and Hedgehog pathways in oxysterol-stimulated PDLSC osteogenic differentiation

**Fig. 5:**
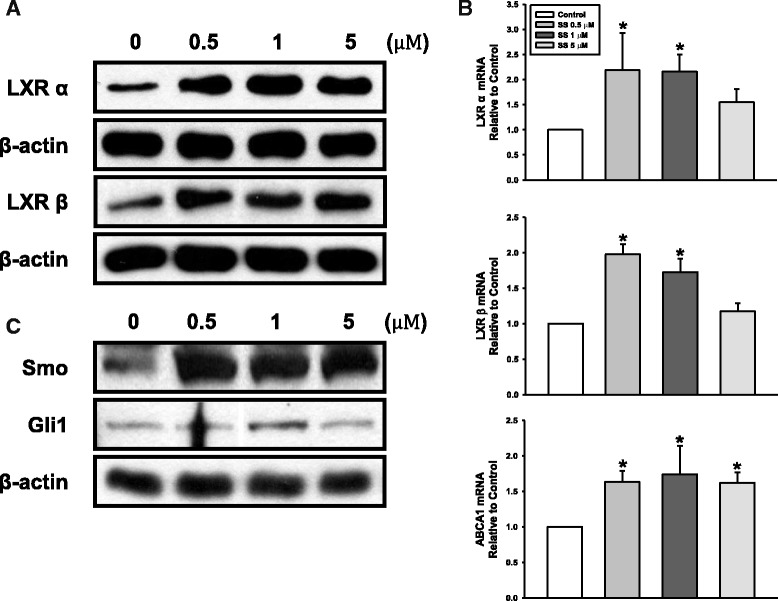
Effect of oxysterols on LXR and Hh signaling. Cells incubated with SS at different concentrations (0.5, 1, and 5 μM). **a** Protein levels of LXRα (50 kDa) and LXRβ (56 kDa). **b** mRNA expression of *LXRα*, *LXRβ*, and *ABCA1*. **c** Protein levels of Smo (85 kDa) and Gli1 (118 kDa). Values reported as means ± SD of four independent experiments. *P < 0.05 vs control value. ABCA1 ATP-binding cassette transporter A1, Hh Hedgehog, LXR liver X receptor, Smo Smoothened

Oxysterols have been known to act as ligands of the LXR nuclear receptors [24]; therefore, we assessed whether these receptors are associated with the osteogenic effect of oxysterols. Western blotting analysis detected increased protein levels of LXRα and LXRβ (isoforms of LXRs) in response to SS treatment (Fig. 5a). In further experiments, we assessed the mRNA expression of *LXRα* and *LXRβ*, and their target gene *ABCA1*, using RT-qPCR. Oxysterol treatment increased the mRNA expression of each gene compared with expression in the control groups (Fig. 5b).

Next, we verified whether oxysterols could activate the Hedgehog (Hh) signaling pathways in PDLSCs. Figure 5c shows that oxysterols augmented the protein levels of the Hh receptor Smo and the transcription factor Gli1, suggesting the involvement of Hh signal transduction in the oxysterol-stimulated osteogenic differentiation of PDLSCs.

### Cross-reaction between LXRs and Hedgehog pathways during PDLSC osteogenesis

**Fig. 6:**
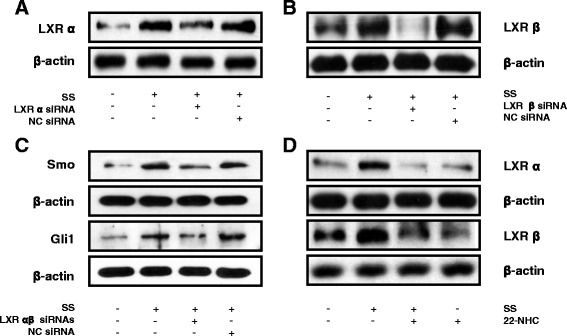
Evaluation of the cross-reaction between LXR and Hh signaling. Cells were transfected with either LXRα or LXRβ-specific siRNAs or a negative control siRNA for 24 h and further incubated with SS (1 μM) for 48 h, and protein levels of (**a**) LXRα and (**b**) LXRβ were assessed. **c** Cells were transfected with both LXRα and LXRβ-specific siRNAs to a final concentration of 100 nM of each siRNA prior to SS treatment, and protein levels of Smo and Gli1 were analyzed. **d** Protein levels of LXRα and LXRβ measured after cells were pretreated with 22-NHC before SS treatment. Representative result from five independent experiments. LXR liver X receptor, NC negative control, 22-NHC 22-azacholesterol, siRNA small interfering RNA, SS 22(S)-hydroxycholesterol and 20(S)-hydroxycholesterol

**Fig. 7:**
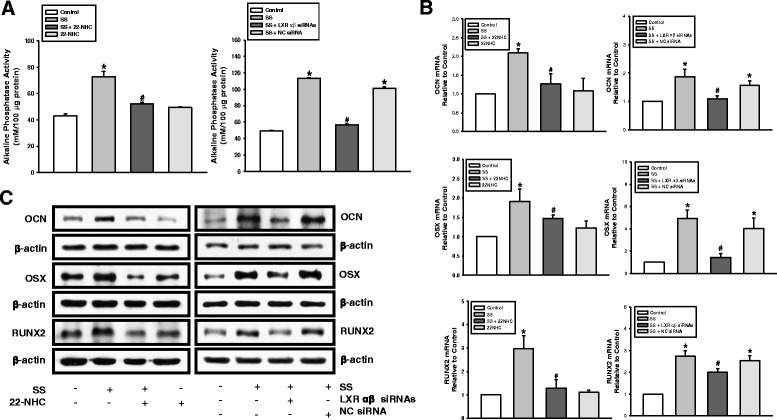
Effect of LXR and Hh signaling on oxysterol-induced PDLSC osteogenesis. Cells pretreated with 22-NHC or transfected with LXRαβ siRNAs before SS (1 μM) treatment. (**a**) ALP activity. (**b**) mRNA expression and (**c**) protein levels of OCN, OSX, and RUNX2. Data obtained from three independent experiments. *P < 0.05 vs control value; ^#^ *P* < 0.05 vs SS treatment alone. LXR liver X receptor, NC negative control, 22-NHC 22-azacholesterol, OCN osteocalcin, OSX osterix, RUNX2 Runt-related transcription factor 2, siRNA small interfering RNA, SS 22(S)-hydroxycholesterol and 20(S)-hydroxycholesterol

To determine the correlation between LXRs and Hh signaling pathways, we performed gene knockdown studies using LXR-specific siRNAs. To validate the efficacy of the *LXRα* or *LXRβ*-specific siRNA, the cells were transfected with either *LXRα*, *LXRβ*, or negative control siRNAs (Fig. 6a, b). In further experiments, knockdown of both *LXRα* and *LXRβ* by siRNAs (*LXR* siRNAs) blocked the increase in Smo and Gli1 protein levels induced by 1 μM SS treatment; however, the negative control siRNA transfection did not affect the oxysterol-induced protein levels (Fig. 6c). Interestingly, pretreatment of the cells with the Smo inhibitor 22-NHC (22-azacholesterol) inhibited the increase in oxysterol-induced LXRα and LXRβ protein levels (Fig. 6d). Although further research is required, these findings provide experimental evidence of a complicated mutual interaction between LXRs and Hh that mediates the oxysterol-induced osteogenic differentiation of PDLSCs. Finally, the pattern of ALP activity and the mRNA expression and protein levels of OCN, OSX, and RUNX2 were also inhibited by either *LXR* siRNAs or 22-NHC treatment (Fig. 7a–c).

### Evaluation of the newly formed bone in the alveolar bone defect site

**Fig. 8:**
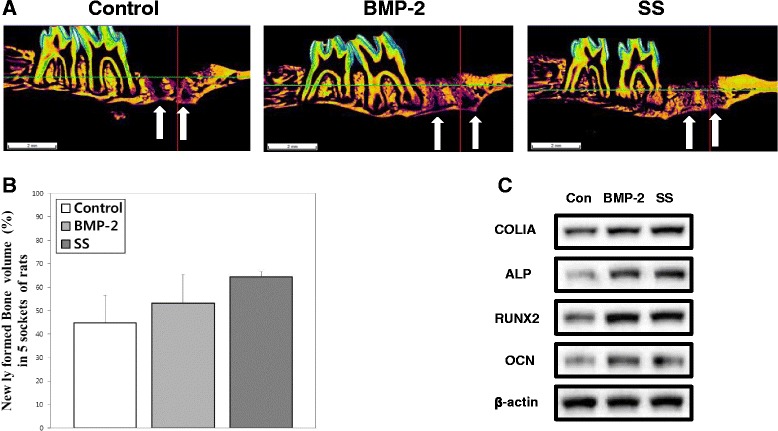
Effect of oxysterols or BMP-2 on alveolar bone regeneration. **a** Color-coded μCT images of extraction sockets in the control, BMP-2, and SS groups 15 days after tooth extraction. Purple area in the sockets (indicated by white arrows) represents newly formed bone (scale bar, 2 mm). **b** Newly formed bone volume in sockets (%) measured three-dimensionally (mean ± SD). **c** Protein levels of COLIA, ALP, RUNX2, and OCN determined to confirm alveolar bone regeneration using western blotting analysis. ALP alkaline phosphatase, BMP‐2 bone morphogenetic protein‐2, Con control, OCN osteocalcin, RUNX2 Runt-related transcription factor 2, SS 22(S)-hydroxycholesterol and 20(S)-hydroxycholesterol

Next, we determined the osteogenic effect of oxysterols on alveolar bone formation. The animal studies were designed as described in Methods. The volume of the newly formed bone in the defect site was initially analyzed using color-coded μCT images. Figure 8a shows the representative color-coded μCT images of the sockets 15 days after the extraction of the maxillary first molars in the control, BMP-2, and SS groups. The purple area in the sockets indicates newly formed bone in the alveolar defect site. In the control group, new bone was formed along the border of the sockets, but the central areas of the sockets were not filled with new bone. In the BMP-2 and SS groups, the sockets were filled with new bone to the alveolar bone crest. When the volume of newly formed bone in the sockets was calculated using three-dimensional analysis software, there were no statistical differences between the three groups (Fig. 8b). However, the new bone volume in the sockets appeared to be higher in the SS group than in the BMP-2 group according to the μCT images. New bone formation was lowest in the control group. These results were also confirmed in the histological images of sockets following H&E staining at 10 and 15 days after extraction and by immunohistochemical staining for OCN and ALP at 15 days after extraction (Additional file 1). In the H&E staining images, new bone and osteoid were growing from the socket walls to the center of the sockets at 10 days in the control group. The BMP-2 and SS groups showed more new bone than the control group at 10 days after the extraction. At 15 days after the extraction, the sockets in the BMP-2 and SS groups were filled with newly formed bone, whereas the center of the sockets in the control group still showed connective tissue surrounded with new bone. The immunohistochemical images also showed higher expression of OCN and ALP in the BMP-2 and SS groups than in the control group at 15 days after extraction. Additionally, we confirmed the alveolar bone regeneration by assessing the protein levels of COLIA, ALP, RUNX2, and OCN using western blotting analysis. As shown in Fig. 8c, the level of each osteogenic-related protein increased considerably in the BMP-2 and SS-treated groups compared with levels in the control group. According to these results, oxysterols and BMP-2 promote alveolar bone regeneration equally.

## Discussion

The present study investigated the potency of oxysterols in the regeneration of periodontal tissue using two methods: assessment of PDLSC activity and alveolar bone reformation. To date, no alternatives to BMP-2 have been developed that are effective in regenerative periodontal therapy. However, we reported previously that certain naturally occurring molecules, such as oxysterols, are osteogenic when applied to ESCs [11]. Based on these findings, the present study first established whether and how a specific combination of oxysterols accelerates the biological activity of PDLSCs. In this study, the novel combination of oxysterols significantly promoted the osteogenic differentiation of PDLSCs. Consistent with our findings, a recent study reported that oxysterol combinations, 22(R)-hydroxycholesterol or 22(S)-hydroxycholesterol and 20(S)-hydroxycholesterol, have a dynamic osteogenic activity [19]. These oxysterol combinations also enhanced osteogenic differentiation at the cellular level and in in-vivo bone healing [13, 18].

Oxysterols exert multiple physiological effects through a variety of cellular receptors, including the LXR nuclear receptors [25, 26]. Although many studies have reported the activity of LXRs in osteogenic regulation, the precise role of these receptors during bone homeostasis has not been determined. Moreover, whether LXR activation influences the physiological performance of PDLSCs is unknown. The present study demonstrated that the combined oxysterol treatment significantly increased the gene and protein expression of *LXRα* and *LXRβ* and their target gene *ABCA1* in PDLSCs during osteogenic differentiation. Conversely, the knockdown of both *LXRα* and *LXRβ* significantly inhibited the oxysterol-induced osteogenic differentiation of PDLSCs. Previous studies also demonstrated that oxysterols, as natural LXR ligands, stimulate the osteogenic differentiation of bone marrow stromal cells [16, 19]. However, paradoxically, treatment with synthetic LXR agonists inhibited the osteogenic differentiation of MSCs [19, 27]. These inconsistent findings suggest that oxysterols might play distinct, yet unidentified, roles in different biological processes. Previous reports have demonstrated the contribution of oxysterol-activated LXRs to bone biology. One of the oxysterol family, 27-hydroxycholesterol, induces LXR activation, which stimulates osteoclasts but inhibits osteoblast function [28]. With regard to osteogenic oxysterols, LXR-independent pathways are suggested to encourage the differentiation of bone marrow stromal cells [19]. In contrast, the long-term activation of LXRs with synthetic LXR agonists has no impact on bone loss, suggesting a biphasic effect of LXRs on bone cell function [29]. Interestingly, the present study showed the beneficial involvement of specific combined oxysterol-activated LXRs in the osteogenic differentiation of PDLSCs. Moreover, the most interesting finding in our study is that LXRs activated by oxysterols interact mutually with Hh signaling during PDLSC osteogenesis.

Hh signaling is known as a crucial regulator of osteoblast differentiation during skeletal development [30]. In particular, osteogenic oxysterols are reported to stimulate Hh signaling to exert osteoinductive effects in in-vitro and in-vivo model systems [11, 18]. Thus, we hypothesized that the osteogenic effect of LXRs on PDLSCs might involve reciprocal action by Hh signaling pathways. Similarly, a previous study demonstrated that LXR activation by osteogenic 20S-hydroxycholesterol cooperates with Hh signaling to induce Notch target genes, playing a significant role in the oxysterol-induced osteogenic differentiation of MSCs [16]. Given the differences in the reports of the function of LXRs in osteogenic biology, the various experimental systems, and their notable role in the osteoinductive effects of oxysterols in vitro, further experiments are required to investigate the specific mechanism by which individual signaling pathways reciprocally enhance the osteogenic process of PDLSCs.

Together with in-vitro PDLSC osteogenesis, our in-vivo study strengthened the concept of the therapeutic effect of oxysterols for periodontal tissue regeneration. Current evidence shows that oxysterols potentially promote osseous repair in alveolar bone defect sites. In particular, when we tried to compare the osteogenic effect between oxysterols and BMP-2, we found that the newly formed bone areas in the oxysterol-injected group appeared larger than the corresponding areas in the BMP-2 group as determined by the μCT images and histological analyses. Using western blotting analysis of the socket specimens, the levels of osteogenic-related proteins in the oxysterol groups were increased compared with the BMP-2 groups. A previous report showed that the semi-synthetic oxysterol Oxy133 has an equivalent efficacy to rhBMP-2 in stimulating spinal fusion [31]. When cranial bone defects were treated with another oxysterol analog, Oxy49, bone regeneration was completed with a comparable efficiency to BMP-2 [32]. Although BMP-2 can result in successful bone regeneration, there are growing concerns regarding the long-term safety and expense of BMP-2 therapy [33, 34]. Thus, in-vivo research suggested the feasibility of oxysterols as agents to regenerate alveolar bone and periodontal tissues in clinical trials.

To date, the regeneration of periodontal tissues remains a complex matter and a challenge in periodontology. From the perspective of reconstructing the damaged periodontal tissues to provide a healthy structure and function, multifaceted studies, including stem cells, therapeutic molecules, and preclinical systems, should be considered to develop and validate research on this intricate issue.

## Conclusion

This study explored whether oxysterols regulated the activity of PDLSCs by a molecular mechanism involving a cross-reaction between the LXRs and the Hh signaling pathway to promote stem cell performance. De-novo stimulation of alveolar bone formation by oxysterols could be of significant clinical interest for periodontal repair, bone fracture regeneration, and the treatment of osteoporosis.

## Additional file

Additional file 1: Figure S1. showing histological assessment of alveolar bone formation. (**A**) H&E staining of the sockets at 10 and 15 days after tooth extraction (40×, 200-μm scale bar on left lower corner). Red dotted lines demarcate the extraction sockets of the maxillary first molar of the rats. Sockets in the BMP-2 and SS groups show more new bone formation than the control group at both 10 and 15 days. (**B**) Immunohistochemical staining of the sockets at 15 days after tooth extraction (200×, 50-μm scale bar on left lower corner). Expression of OCN and ALP is higher in the BMP-2 and SS groups than in the control group. All procedures performed according to standard methods (PDF 1139 kb)

## Abbreviations

22-NHC: 22-Azacholesterol
ABCA1: ATP-binding cassette transporter A1
ALP: Alkaline phosphatase
BMP‐2: Bone morphogenetic protein‐2
COLIA: Collagen type IA
ESC: Embryonic stem cell
Hh: Hedgehog
LXR: Liver X receptor
MSC: Mesenchymal stem cell
OCN: Osteocalcin
OPN: Osteopontin
OSX: Osterix
PDLSC: Periodontal ligament stem cell
RUNX2: Runt-related transcription factor 2
Smo: Smoothened
SS: 22(S)-hydroxycholesterol and 20(S)-hydroxycholesterol
