# Supplementary figures and images for: Evaluating the oxysterol combination of 22(S)-hydroxycholesterol and 20(S)-hydroxycholesterol in periodontal regeneration using periodontal ligament stem cells and alveolar bone healing models
Source: Stem Cell Res Ther. 2017 Dec 6;8:276. doi: 10.1186/s13287-017-0725-9 (PMC5717822; doi:10.1186/s13287-017-0725-9)

**A**

**Control**

**BMP-2**

**SS**

**10 days**

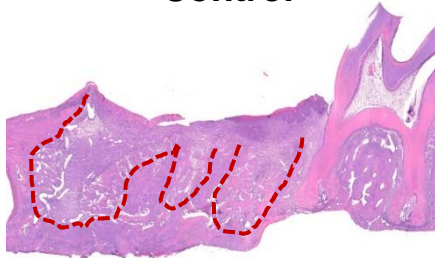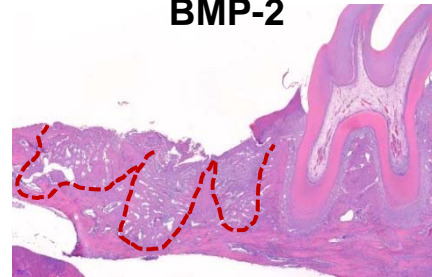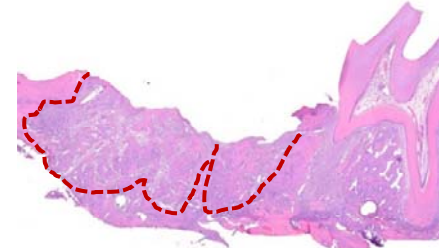

**15 days**

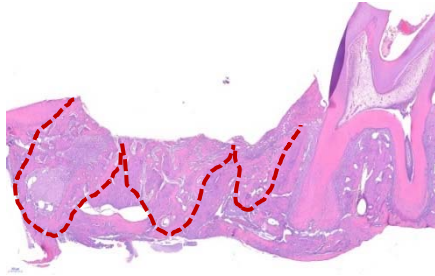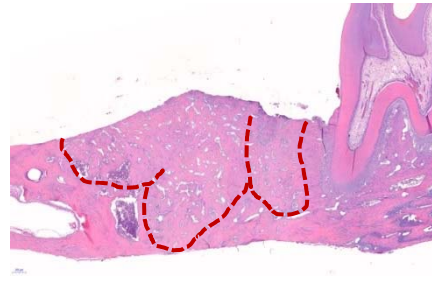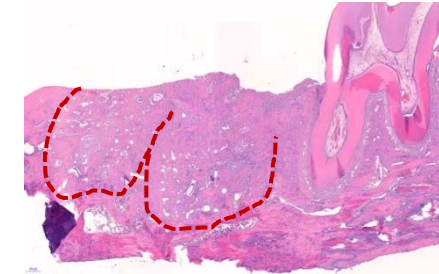

**B**

**OCN**

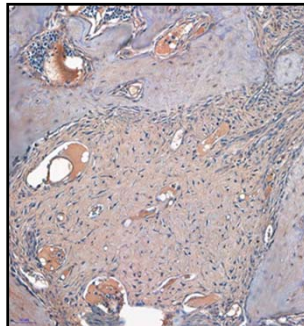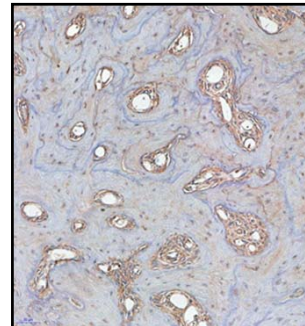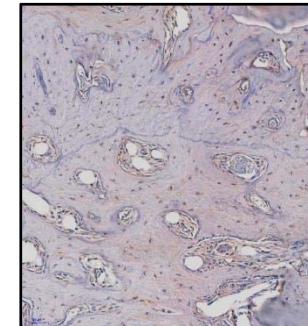

**ALP**

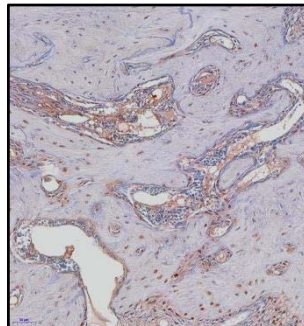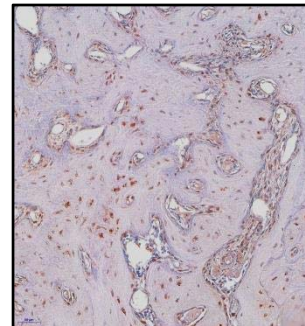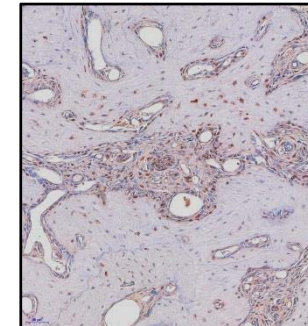

Supplement: Additional file 1: Figure S1. — showing histological assessment of alveolar bone formation. (A) H&E staining of the sockets at 10 and 15 days after tooth extraction (40×, 200-μm scale bar on left lower corner). Red dotted lines demarcate the extraction sockets of the maxillary first molar of the rats. Sockets in the BMP-2 and SS groups show more new bone formation than the control group at both 10 and 15 days. (B) Immunohistochemical staining of the sockets at 15 days after tooth extraction (200×, 50-μm scale bar on left lower corner). Expression of OCN and ALP is higher in the BMP-2 and SS groups than in the control group. All procedures performed according to standard methods (PDF 1139 kb) [file 13287_2017_725_MOESM1_ESM.pdf]
